# Why We Disagree about the Climate Impact of Forestry – A Quantitative Analysis of Swedish Research

**DOI:** 10.1007/s00267-025-02208-z

**Published:** 2025-06-24

**Authors:** Göran Englund, Jeannette Eggers, Bengt-Gunnar Jonsson, Maximilian Schulte, Torbjörn Skytt

**Affiliations:** 1 Umeå University, Department of Ecology, Environment and Geoscience, Umeå, Sweden; 2 Swedish University of Agricultural Sciences, Department of Forest Resource Management, Umeå, Sweden; 3 Mid Sweden University, Department of Natural Sciences, Design and Sustainable Development, Sundsvall, Sweden; 4 Swedish University of Agricultural Sciences, Department of Wildlife, Fish & Environmental Studies, Umeå, Sweden; 5 Swedish University of Agricultural Sciences, Department of Energy & Technology, Uppsala, Sweden; 6 Wageningen University and Research, Wageningen Environmental Research (WENR), Wageningen, The Netherlands; 7 Mid Sweden University, Department of Ecotechnology & Sustainable Building, Östersund, Sweden

**Keywords:** Forestry, Climate impact, Scientific dispute, Substitution, Reduced harvest, Intensified forest management

## Abstract

Intensifying forest management or reducing harvest levels are proposed as alternative strategies for mitigating climate change. Today, scientific disagreement over which approach is more effective impedes the development and implementation of effective climate change mitigation policies. In this paper we review studies of the climate impact of Swedish forestry to clarify the conceptual and methodological differences that underly the disagreement. To examine how assumptions concerning crucial parameters contribute to differing conclusions, we simulated various management scenarios for Gävleborg County in central Sweden. We find that support for either side in the debate can be obtained by adjusting assumptions about substitution levels and the design of management interventions. Studies favoring intensified management over reduced harvesting assume relatively high substitution levels and implement intervention levels — such as increased fertilization or expanded stump harvest — which are considerably higher (2.4–17.7 times) than the levels recommended by the Swedish Forest Agency. Conversely, when using recommended intervention levels and substitution levels based on current usage of forest biomass, reduced harvest strategies show greater climate benefits than intensified management. These findings emphasize the need to focus the scientific discussion on i) the empirical evidence for various substitution levels and ii) the relevance of alternative management scenarios for the development of effective climate change mitigation policies.

## Introduction

As humanity strives to decarbonize the global economy and reduce reliance on fossil fuels, the importance of renewable materials grows. Forest-based products and fuels could play a crucial role in this shift. Yet, the scientific community remains divided over the climate impacts of various forest management strategies. Some studies point in the direction that intensive rotation forestry in combination with effective substitution maximizes the climate benefits that can be obtained from the forest sector (Gustavsson et al. 2021; Petersson et al. 2022; Schulze et al. 2022; Jandl et al. 2024), whereas others suggest that reducing harvest levels provides greater climate benefits than today’s intensive forestry in the next 50–100 years (Braun et al. 2016; Schulte et al. 2022; Soimakallio et al. 2022; Peng et al. 2023). Interestingly, there is widespread agreement that the forest sector can provide considerable climate change mitigation. This consensus implies that resolving the dispute will have major policy implications and ultimately improve our ability to mitigate global warming. Thus, it is important to gain a better understanding of the basis for this dispute.

Disagreement and a plurality of ideas are important for scientific progress (Shaw 2021), but may compromise the credibility of science in the eyes of the public and policymakers (Chinn and Hart 2022), and impede policy development (Oreskes and Conway 2010). Scientific disputes are sometimes resolved when one side accumulates enough supporting evidence to convince the scientific community (e.g., Sudiro 2014). However, this is typically a slow process that plays out over decades, and a potentially more rapid road to resolution is to perform detailed comparative analyses of the methods, definitions, models, and data used (Latham et al. 1988, Abrams and Ginzburg 2000; Strengers 2024). A complication when analysing studies of the climate impact of forestry, is that they differ in a wide range of aspects, making it difficult to pinpoint which aspects are quantitatively important for the conclusions reached. A related problem is that the reporting of methods and assumptions rarely is detailed enough to allow replication of the findings.

The situation is, however, improving as there is growing consensus about methods, assumptions and reporting. Several recent analyses of the climate impacts of Swedish forestry have been based on the same national-level forest data (Fridman et al. 2014), the same simulation tool for forest carbon dynamics (i.e., the Heureka software, Lämås et al. 2023), and the same models describing the dynamics of the carbon stored in harvested wood products (IPCC 2019). Moreover, the reporting of methods and assumptions are often detailed enough to reveal important differences. Thus, it seems that the scientific debate about the climate impact of Swedish forestry provides an excellent opportunity to clarify “why we disagree”.

In this study, we first perform a detailed review of the literature to identify important conceptual and methodological differences between studies. On the basis of the review, we identify two types of assumptions that could contribute to the disagreement: i) the substitution levels assumed, and ii) the choice of alternative scenarios that today’s forest management is compared with. On the basis of these findings, we then perform quantitative analyses to demonstrate how the different assumptions affect the estimated climate impact of Swedish forestry.

## Review of Studies on the Climate Impact of Swedish Forestry

We identified eight peer-reviewed studies published between 2014 and 2023 that examined the climate impact of Swedish forestry using the simulation package Heureka or its predecessor Hugin. They all quantify changes in the carbon stored in forest and harvested wood products, and emissions avoided through substitution. The net effect of such changes that reduces the concentration of CO_2_ in the atmosphere we will henceforth be referred to as a positive climate impact. The effect of the current forest management can be evaluated by comparing the climate impact of today’s forestry and the impact that would be seen with no harvest and no other management actions. Only one study included a no-harvest scenario (Skytt et al. 2021), and a more common approach was to examine the effects of increasing or decreasing harvest levels by 10–40% (Gustavsson et al. 2017; Lundmark et al. 2018; Gustavsson et al. 2021; Skytt et al. 2021; Petersson et al. 2022; Schulte et al. 2022). Three different methods have been used to modify harvest levels: i) increasing the area set aside as reserves (Gustavsson et al. 2017; Gustavsson et al. 2021; Petersson et al. 2022), ii) changing the rotation period (Lundmark et al. 2018; Schulte et al. 2022), or iii) changing the proportion of yearly growth that is harvested, including both thinning and final felling (Skytt et al. 2021; Schulte et al. 2022). The general result from these studies is that reduced harvest has a positive climate impact for 50–200 years or more. However, in an even longer perspective, the impact becomes negative (Gustavsson et al. 2017; Skytt et al. 2022). These findings appear to be widely accepted (Eggers and Schulte 2023; Rummukainen 2024), but a major disagreement remains concerning the relative importance of short-term and long-term effects (Gustavsson et al. 2022; Skytt et al. 2022).

Several studies have asked whether the climate impact of today’s forestry can be enhanced by more intensive management, e.g., planting fast-growing species, increasing the use of fertilizers (Lundmark et al. 2014; Gustavsson et al. 2017; Petersson et al. 2022) and increasing the harvest of slash and stumps (Lundmark et al. 2014; Cintas et al. 2017; Gustavsson et al. 2017; Gustavsson et al. 2021). The general finding is that more intensive management has a positive effect on the climate.

Thus, the literature describes two different strategies that can increase the climate benefit of forestry: reducing harvests or intensifying management. A key question is then which of the two strategies is more beneficial for climate change mitigation. Three studies have addressed this question by comparing a scenario where the area set aside as reserves is increased to scenarios with intensified management (Gustavsson et al. 2017; Gustavsson et al. 2021; Petersson et al. 2022). These studies find that increasing the area set aside provides a comparably small benefit over the next 20-40 years and that intensified management provides much greater benefits in a longer time perspective.

This short review suggests that the scientific controversy, at least to some extent, occurs because the problem often is defined differently by the two sides. Some studies argue that decreased harvest levels would provide considerable climate benefits during the coming decades. Other studies argue that intensified management has the potential to provide greater climate benefits in the long term.

A second conclusion that we draw is that there is a need for more comprehensive analyses of the relative benefits of intensive management and reduced harvest. Studies that include a comparison of the two strategies (Gustavsson et al. 2017; Gustavsson et al. 2021; Petersson et al. 2022) assume comparably high substitution levels (Table 1) and study scenarios with intervention levels that in some cases are in conflict with the Swedish Forestry Act and its general guidelines. As these aspects may affect the conclusions reached we will in the next two sections review and discuss substitution assumptions and choices of intervention levels made in the different studies.

**Table 1:** Methods used in the reviewed studies

Study	Spatial extent	Temporal extent	Scenarios studied	Realized substitution level (ton fossil C subst/ton C harvested)	Displacement factor (ton fossil C/ton C in wood products)	Proportion of harvest that provides substitution	Climate effect on tree growth	Extraction of harvest residues in reference	Harvest level in reference (% of net growth)	Half-lives for harvested wood products (or life spans)
Schulte et al. (2022)	3 counties in southern, central and northern Sweden Sweden)	200 y	Harvest levels, rotation length	0.52	0.6	54%	No	Yes	83%	Sawn wood = 35 y, boards = 25 y, paper and pulp = 2 y, bioenergy = 1 y
Petersson et al. (2022)	Sweden	200 y	Increased area set aside, increased fertilization	1.30	1.30	100%	No (but evaluated in a “positive climate effect” scenario)	Yes (tops and branches corresponding to 10 TWh	~100%	Sawn wood = 35 y, boards = 25 y, paper and pulp = 2 y
Skytt et al. (2021)	5 counties along a north-south gradient	150 y	Harvest levels	0.65	0.95	44%	No	No	80%	Sawn wood = 35 y, boards = 25 y, paper and pulp = 0 y, bioenergy = 0 y
Gustavsson et al. (2021)	1 county in southern Sweden	200 y	Increased area set aside, increased growth (fertilization, species selection), increased use of harvest residues	Not reported	Not reported	Not reported	Yes (RCP 4.5)	No	~100%	80 y life span for buildings
Lundmark et al. (2018)	Single model stands	One rotation	Prolonged rotation period	Not reported	Not reported	Not reported	Not reported	Yes (60% of tops and branches)		Not reported
Gustavsson et al. (2017)	Sweden	100 y	Increased area set aside, increased growth (fertilization, planting fast growing species), increased use of harvest residues.	0.9–1.1^a^	Not reported	Not reported	Yes (RCP 4.5)	Yes (tops and branches corresponding to 8 TWh)	~100%	80 y life span for buildings
Cintas et al. (2017)	Sweden	90 y	Increased harvest of residues, increased fertilization, intensified regeneration	Not reported	2.5 for sawn wood, 0.6 for bioenergy^b^	not reported	No	Yes, 15% of harvest residues	~100%	Sawn wood = 35 y, boards = 25 y, paper and pulp = 2 y
Lundmark et al. (2014)	Sweden	95 y	Increased harvest of residues, increased fertilization and intensified regeneration	0.42^c^	Not reported	Not reported	No	Yes, 15% of harvest residues	~100%	Life spans e.g.: buildings 80 y, furniture 30 y, bioenergy 2 y.

^a^Deduced substitution level required to reproduce the climate benefits reported in Fig. 9 in Gustavsson et al. (2017) (calculations given in the Supplementary Material)

^b^Concerns export; all sawn wood substitute concrete

^c^Substitution level calculated based on data in Fig. 3c in Lundmark et al. (2014), (calculations given in Supplementary Material)

### Assumptions about Substitution

Emissions that are avoided when fossil-intensive products are substituted with biomass-based products is an important component when calculating the climate impact of forest management. The effects of substitution are often described using displacement factors (DF) (Sathre and O’Connor 2010), which quantify the emissions of greenhouse gases that are avoided if a fossil-based product is replaced by a biomass-based alternative. The total substitution effect of all different wood products is quantified with an aggregated displacement factor. This factor is calculated as the weighted average of all product-specific displacement factors, using as weights each product’s share of the total usage of the harvested biomass (Hurmekoski et al. 2021; Skytt et al. 2021; Schulte et al. 2022). We will refer to this type of factor as a usage-based or market-level displacement factor (Hurmekoski et al. 2021).

The realized substitution effect is also affected by how large share of the harvest that is used for substitution. While some studies assume that 100% of the extracted biomass replaces fossil products, others have argued that some product groups, such as graphic paper and many paperboard products, lack fossil-based alternatives and thus cannot provide substitution benefits (other than end-of-life incineration) (Lundmark et al. 2014; Petersson et al. 2022).

The substitution assumptions made in the reviewed studies are presented in Table 1. Displacement factors were reported in only four of the eight studies. For this reason, we focus on the realized substitution level, which is the total substitution benefit expressed as a fraction of the total amount of carbon harvested (unit: ton C substituted/ton C harvested). This measure has the additional advantage that it incorporates assumptions about the harvest’s share that provides substitution. If the substitution level was reported in the unit ton C/m^3^ wood it was transformed to the unit ton/ton (e.g., Petersson et al. 2022), using 1.3 as conversion factor. For studies using three levels of displacement (high, intermediate, and low values) (Skytt et al. 2021; Petersson et al. 2022; Schulte et al. 2022), we focused on the intermediate value, assuming that it represents the authors best estimate. When substitution levels were reported for several different scenarios we focused on the base-line scenario (e.g., Lundmark et al. 2014). Three studies, Lundmark et al. (2014), Skytt et al. (2021) and Schulte et al. (2022), estimated substitution on the basis of the actual usage of the harvested biomass and reported realized substitution levels ranging from 0.42 to 0.65 (ton C substituted/ton C harvested). The realized substitution levels used in Petersson et al. (2022) and Gustavsson et al. (2017) was higher (0.9–1.3). Petersson et al. (2022) did not provide details about how the substitution level was determined. Gustavsson et al. (2017) did not report substitution levels, but based on data supplied by the authors we could estimate that the effect of substituting concrete frame buildings and fossil fuels corresponded to an overall realized substitution level of 0.9–1.1, depending on which scenario was studied. These values were not reported by the authors but are deduced values required to reproduce reported climate benefits when using the modelling framework applied in this paper. Gustavsson et al. (2021) used methods similar to those of Gustavsson et al. (2017), suggesting comparable substitution levels. Finally, the realized substitution levels of Lundmark et al. (2018) and Cintas et al. (2017) were not reported and could not be estimated. Calculations of estimated substitution levels in Lundmark (2014) and Gustavsson (2017) are reported in the supplementary material.

### Magnitude of Interventions

The outcome of comparisons between different management strategies, e.g., intensified management and reduced harvest, will, for obvious reasons, depend on the level of intervention. For example, a small decrease in the harvest level will likely provide smaller climate benefits than a large increase in the fertilized area, and vice versa. Although it is often of interest to investigate extreme intervention levels, e.g., a no harvest scenario, we propose that for a comparative study to be relevant for policy makers, it should also include interventions that represent an acceptable maximal potential based on current economic, ecological, and social considerations. Categorizing interventions on the basis of their realism is challenging, as what is considered acceptable will likely vary among researchers. Nevertheless, it may be necessary to agree on this issue to reach a consensus. Thus, it is important to discuss what levels of intervention represent an acceptable maximum potential. Such discussions are already present in the grey literature, in the form of consensus processes led by the Swedish Forest Agency (Skogsstyrelsen) that are meant to balance economic and environmental arguments, as stated in the Forestry Act, while also accounting for practical limitations (e.g., Skogsstyrelsen 2018). The outcome is recommendations specifying an “acceptable maximum potential” for different management actions. For fertilization, a threefold increase in the fertilized area (to 100,000 ha/year) is recommended (Skogsstyrelsen 2018). For extraction of stumps it is recommended that it should be limited to 5–10% of the harvested area and that deciduous stumps and 20% of coniferous stumps should be left (Skogsstyrelsen 2009). For planting the non-native species *Pinus contorta*, the maximal area stated in the Swedish Forestry Act is 14,000 ha/year. For extraction of slash, the Swedish Forestry Research Institute recommends that biomass corresponding to 21 TWh, which is 2.3 times the present level, can be extracted (Skogforsk 2023), (see also de Jong et al. (2017). We propose that these levels can be seen as acceptable maximal potentials.

Three published studies compared the climate benefits of intensified management and reduced harvest (Gustavsson et al. 2017; Gustavsson et al. 2021; Petersson et al. 2022). The levels of intervention in the intensified management scenarios were considerably higher (2.4–17.7 times) than the levels recommended as acceptable maximum potential (Table 3). This motivated us to investigate whether the outcome of comparisons between the two major strategies changes if the levels of intervention are limited to recommended levels.

For reduced-harvest strategies, there are no clear recommendations specifying an acceptable potential. The harvest intensity in forest land managed for wood supply has increased during the last 10 years, from an average of ~73% of the annual net growth during the period 2000–2015 to nearly 100% today (Roberge et al. 2024). Thus, we propose that reducing the harvest level to 75% of the available growth could be considered an acceptable potential. A similar argument can be made for prolonging the rotation period, where a 30-year prolongation produces a similar reduction in the average harvest over the first 50 years. But note that the reduction is larger in a shorter perspective and smaller in a longer perspective. For increasing the area set aside from forestry, two studies assumed a doubling of the area set aside (Gustavsson et al. 2017; Petersson et al. 2022), whereas Gustavsson et al. (2021) assumed an increase with a factor of 4.5. According to the Swedish Environmental Protection Agency, 30% of the Swedish land area should be given “formal area-based protection and other area-based effective protection measures” until 2030 (Naturvårdsverket 2023). This is based on international agreements (EC 2020; CBD 2022). As setting aside 30% of the productive forest would increase the protected area approximately three times, we propose that a tripling of the area set aside could be seen as an acceptable potential. We caution, however, that the international agreements have not yet been implemented, and it is possible that some closer-to-nature-forestry will be allowed in protected forests. Thus, we also present results for a doubling of the set-aside area.

## Quantitative Analyses

The aim of the quantitative study is to demonstrate how substitution assumptions and choices of alternative scenarios affect estimated climate benefits. Our overall approach is to incorporate different assumptions in a modelling framework under *ceteris paribus* conditions, i.e., by keeping everything else equal we can study the effect of a specific assumption on the estimated climate benefit. This was accomplished by applying different assumptions in simulations of forestry in Gävleborg county, central Sweden. Gävleborg was chosen because many aspects of its forests can be considered representative of national average conditions (Table 2). There are 1,486,000 ha productive forest land (productivity >1 m^3^ ha^−1^ year^−1^) that is dominated by *Pinus sylvestris* (50%, based on volume), *Picea abies* (33%) and *Betula spp*. (12%) (NFI 2024).

**Table 2:** Forest data for the Gävleborg county and Sweden, which are based on the national forest inventory for the period of 2014–2018 (NFI 2024)

Property	Gävleborg	Sweden
Productive forest area (kha)	1486	23,549
Mean volume (m^3^ ha^−1^)	149.6	141.1
Average productivity (m^3^ ha^−1^ year^−1^)	5.53	5.33
Mean age (year)	51.7	62.5
Mean annual temperature (°C)	5.0	4.9

Temperature data are provided by SMHI (2022)

The procedure used to calculate the climate impact of the different management strategies and substitution assumptions involved the following steps:

- Simulate forest growth, mortality and decomposition of deadwood for different management scenarios to estimate changes in total forest carbon stock, including living trees, dead trees, and soil.
- Map the current wood flow from harvest to the final product to estimate changes in the carbon stored in wood products.
- Estimate realized substitution levels. This quantity specifies avoided carbon emissions as a fraction of the harvested amount of carbon. For reduced harvest scenarios it accounts for the increased use of fossil-based fuels and products.
- For each management alternative, calculate how the total carbon balance change over time, including substitution effects and changes in the amount of carbon stored in forests and products.

### Forest Modelling

The future development of the carbon stored in living trees, dead trees and soil was simulated for Gävleborg county over 150 years, using the RegWise module in the Heureka package (Lämås et al. 2023). This simulation tool is widely used in analyses of Scandinavian forestry by researchers, forest owners, and the forest industry (Lämås et al. 2023). Data from the Swedish National Forest Inventory 2014–2018 were used to specify initial conditions such as species composition, forest age, and site productivity (NFI 2024). For Gävleborg, these data are based on 2127 sample plots. The choice of simulation period is crucial and a detailed motivation for choosing 150 years is outlined in the discussion.

As reference, we used a business-as-usual scenario (henceforth BAU) that mimics the dominant management strategy in Sweden. This means harvest through clear-cutting, followed by regeneration, cleaning, and thinning. The harvest level was set to 95% of the annual net growth on all land, which corresponded to 98% of the growth on timber-producing land. This corresponds to the harvest levels used in a majority of the reviewed studies (Table 1) and it is similar to the present harvest level in Sweden (Roberge et al. 2024). The fraction of forest area fertilized each year was set to 0.14% (Skogsstyrelsen 2022), and the dose was 150 kg N/ha. The NFI data for Gävleborg County included a relatively small area of reserves (1%). Thus, to increase the area with formal or voluntary protection to a value closer to the national average (11.5%, excluding retention patches, Statistics Sweden (2022)), stands older than 116 years were set aside for conservation, which increased the area to 10.3% of the productive forest area. The yearly extraction of harvest residues, in the form of tops, branches and needles, was set to 0.078 tons dry matter ha^−1^, which is equivalent to the national average value for 2018–2022 (Statistics Sweden 2022). Regeneration was performed mainly through planting seedlings, following the scenario “todays forestry” in SKA22 (Skogsstyrelsen 2022). All other parameters were set to default values provided in Heureka.

We compared the reference scenario with alternative management scenarios that were selected to illustrate how different conclusions could be reached. In a first set of analyses we examined the effect of varying substitution level for five different scenarios: Reduced harvest level, longer rotation period, increased area set aside, increased area fertilized, and increased slash harvest. The levels used in the different scenarios are given in Table 3, and followed the recommendations described above in the section “*levels of intervention”*.

**Table 3:** Assumed intervention levels for the different management actions applied in the analysed scenarios for Gävleborg County

Scenario	Target harvest level (% of growth)	Realized harvest (m^3^fub year^−1^ ha^−1^)	Rotation period (y)	Area set aside (%)	Fertilized area (ha year^−1^)	Slash harvest (ton dry matter ha^−1^ year^−1^)
Business as usual (BAU)	95	4.20	78	10.3	2100	0.078
Reduced harvest level	75	3.24	107	10.3	2100	0.078
Longer rotation period	95	3.71	109	10.3	2100	0.078
Doubling area set aside	95	3.73	73	20.6	2100	0.078
Tripling area set aside	95	3.19	77	30.9	2100	0.078
Increased fertilization	95	4.23	78	10.3	6300	0.078
Increased slash harvest	95	4.95	78	10.3	2100	0.180

The levels realized in the simulations varied over time. Thus, we report mean values for the simulated period (150 years)

In a second set of analyses we replicated major assumptions made in two of the studies that compared reduced harvest and intensified management (Petersson et al. 2022; Gustavsson et al. 2017). In both studies, the reduced-harvest scenario was a doubling of the area set aside as reserves, which was contrasted with a high-fertilization scenario in Petersson et al. (2022), and a high production scenario in Gustavsson et al. (2017). Petersson et al. (2022) reported in the methods section that the fertilized area was increased 7 times over the reference scenario. However, the actual level used was increased nearly 35 times. We included both levels in our study. The high production scenario involved interventions meant to increase growth and biomass harvest (e.g., increased fertilization, slash harvest, stump harvest, plantation of nonnative *Pinus contorta*). The intervention levels used in the two studies are provided in Table 4. We emphasize that perfect replication of the results presented by Gustavsson et al. (2017 and Petersson et al. (2022) could not be achieved as the simulation model Heureka has been updated, and we did not have access to all details concerning the parameterization of the Heureka model. As alternatives to the intervention levels used in Petersson et al. (2022) and Gustavsson et al. (2017) we used recommended maximum levels (see section *Magnitude of intervention*s above). Detailed accounts of the calculations of intervention levels, parameterization of the Heureka simulation model, and output data are provided in the supplementary material.

**Table 4:** Intervention levels used when reproducing the assumptions made in different scenarios in Petersson et al. (2022) and Gustavsson et al. (2017)

Intervention	BAU scenario	Increased set-aside scenario^a, b^	Increased fertilization scenario^a^	High production scenario^b^	Recommended maximum intervention levels
Area set aside (%)	10.3	20.6	10.3	10.3	30.9
Fertilized area (ha year^−1^)	2100	2100	14,700, 73,500	4200	6300
Slash harvest (ton dry matter ha^−1^ year^−1^)	0.078	0.078	0.078	0.43	0.18
Stump harvest (ton dry matter ha^−1^ year^−1^)	0	0	0	0.23	0.013
Planting *Pinus contorta* (ha year^−1^)	0	0	0	5359	1504
Realized harvest (m^3^fub year^−1^ ha^−1^)	4.20	3.73	5.01, 6.00	4.39	–

For comparison we provide the recommended maximum intervention levels. BAU refers to the business as usual scenario. We also report the realized harvest levels in the different scenarios

^a^Used in Petersson et al. (2022)

^b^Used in Gustavsson et al. (2017)

### Modelling Substitution and Carbon in Wood Products

In our first set of analyses we estimated the climate impact of three different levels of realized substitution (RS), corresponding to the levels used in Lundmark et al. (2014) (RS = 0.42), Skytt et al. (2021) (RS = 0.65), and Petersson et al. (2022) (RS = 1.30). In the second set of analyses we contrasted the estimated climate impact when assuming either a market-level substitution level based on the usage of forest biomass, or the levels used in Gustavsson et al. (2017) and Petersson et al. (2022). As an estimate of realized market-level substitution level, we used RS = 0.53, which is the mean of the values presented in Lundmark et al. (2014), Skytt et al. (2021), and Schulte et al. (2022). This value is in close agreement with the mean value reported in a meta-analysis by Hurmekoski et al. (2021).

In all analyses we modelled the amount of carbon stored in wood products using input/output models and half-life values recommended by IPCC (2019): sawn wood = 35 years, boards = 25 years, paper and pulp = 2 years. Biofuels were assumed to be consumed within five years, which is also the temporal resolution of the Heureka simulations. The fractions of the harvested volume that ended up in different types of products follow Schulte et al. (2022). Calculations of substitution effects, product pool dynamics and total climate benefits are given in the supplementary material.

## Results

**Fig. 1:**
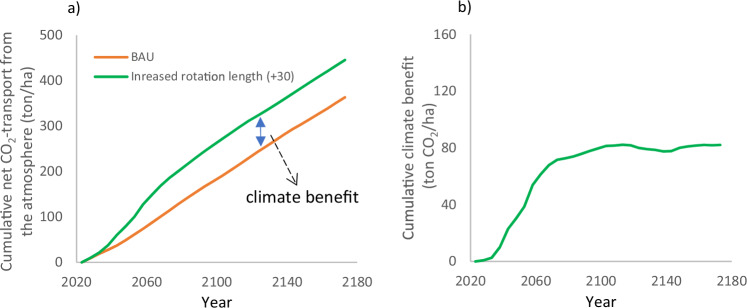
**a** The cumulative carbon uptake of a business-as-usual scenario (BAU) and a scenario with increased rotation length. **b** The climate benefit of the scenario with increased rotation length (+30 years). Climate benefit is given by the difference between the CO_2_-uptake in the BAU scenario and the scenario with increased rotation length (indicated by the blue arrow in (**a**). Realized substitution is 0.65 ton C/ton C

To illustrate how the climate impact of an alternative scenario develops over time, we present the difference in carbon uptake between the business-as-usual (BAU) scenario and the alternative scenario (indicated by the blue arrow in Fig. 1a). In this example the alternative scenario is increased rotation length. This uptake includes the effects of substitution as well as changes in carbon stocks in living and dead trees, soil, and wood products.

Figure 1b shows the difference between the BAU scenario and the alternative scenario. A positive value in this figure indicates that the scenario with longer rotations results in greater net carbon uptake than the BAU scenario. Throughout the text, we refer to the difference between the BAU scenario and an alternative scenario as the climate benefit of the alternative scenario.

### Effects of the Level of Substitution

**Fig. 2:**
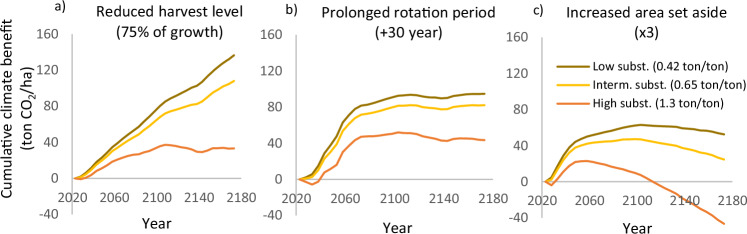
Effects of realized substitution level on the cumulative climate benefit for three different scenarios with decreased harvest. **a** The harvest was reduced from 95% to 75% of annual growth. **b** The rotation period was prolonged with 30 years. **c** The area set aside as reserves was increased by a factor three. The realized substitution levels (RS) correspond to those used in Lundmark et al. (2014), RS = 0.42, Skytt et al. (2021), RS = 0.65 and Petersson et al. (2022), RS = 1.30. The unit for the realized substitution level is ton avoided C emissions/ton C harvested

**Fig. 3:**
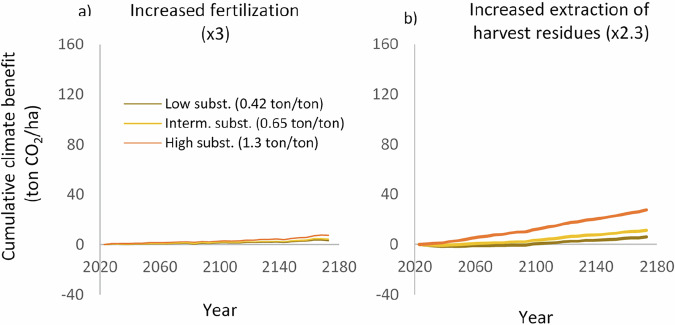
Effects of the assumed level of substitution on the climate impact of intensified management. **a** Shows the effect of increasing the fertilized area three times, whereas **b** illustrates the effect of increasing extraction of harvest residues 2.3 times

The chosen level of substitution strongly affects the estimated climate benefits of different management scenarios (Figs. 2 and 3). Two aspects of the simulation results are particularly relevant to the ongoing debate.

First, substitution affects reduced harvest and intensified management strategies in opposite ways. For intensified management (Fig. 3), higher substitution levels lead to greater climate benefits. In contrast, for reduced harvest scenarios (Fig. 2), the climate benefit decreases as substitution increases. This means that the relative advantage of intensified management grows with higher substitution levels.

**Fig. 4:**
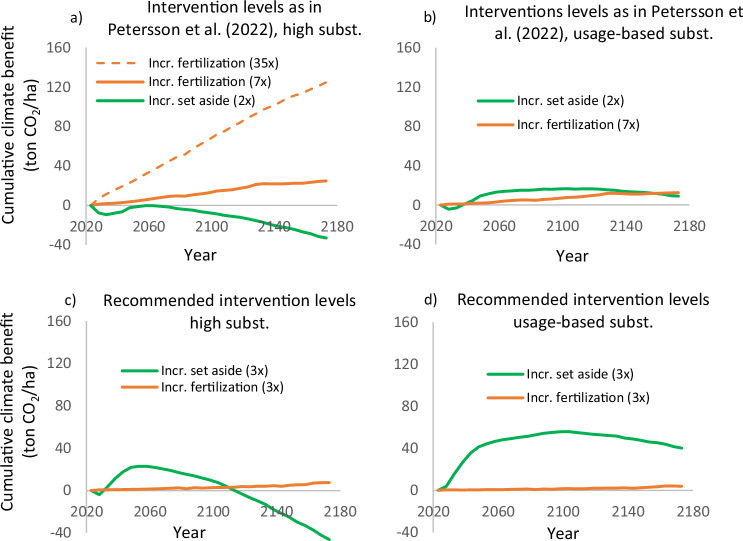
The climate benefit of reduced harvest and increased fertilization when **a** using realized substitution level (1.3 ton/ton) and intervention levels as in Petersson et al. (2022), **b** lowering the substitution level to usage-based value (0.53 ton/ton), **c** retaining the high substitution level but using recommended intervention levels, and **d** using both usage-based substitution and recommended intervention levels. The factors given in the legends (35×, 7×, 2×, 3×) indicate how many times intervention levels were increased over the levels used in the business as usual scenario

**Fig. 5:**
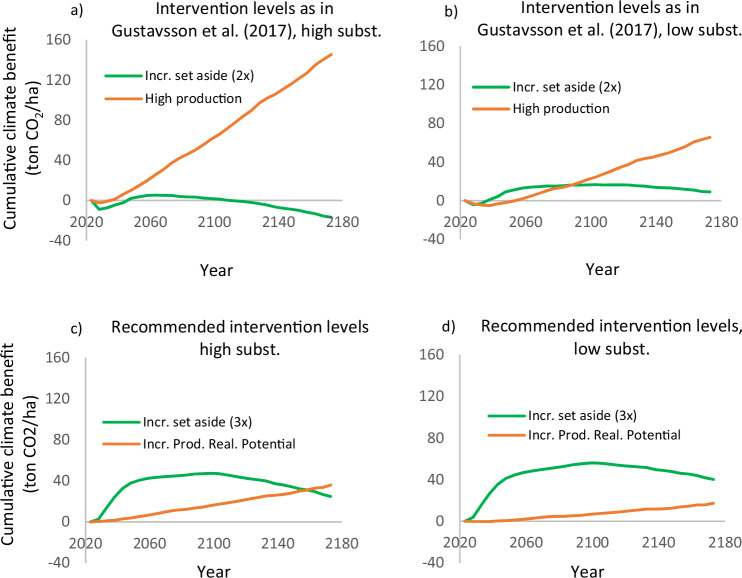
The climate benefit of reduced harvest and the high production scenario used in Gustavsson et al. (2017), when **a** using realized substitution level (1.0 ton/ton) and interventions levels as in Gustavsson et al. (2017), **b** lowering the substitution level to the usage-based value (0.53 ton/ton), **c** retaining the high substitution level but using recommended intervention levels, and **d** using both usage-based substitution and recommended intervention levels. The factors given in the legends (2×, 3×) indicate how many times intervention levels were increased over the levels used in the business as usual scenario

Second, the climate benefit of reduced harvest scenarios may be limited in time. As shown in Fig. 2c, setting aside more forest area initially provides climate benefits, but with high substitution, these benefits become negative after about 100 years. If the set-aside area is smaller—such as a doubling of the current area—the positive effect is even less pronounced or absent altogether (Figs. 4a and 5a; Supplementary Fig. S1). These findings support the argument made by Gustavsson et al. (2017) and Petersson et al. (2022) that reduced harvest may not be an effective strategy for climate change mitigation. However, for the other scenarios in Fig. 2a–c, the climate benefit remains positive over the next 150 years, although negative values may occur over longer time periods.

### Comparisons of Reduced Harvest and Intensified Management

As shown in Figs. 2 and 3, the relative climate benefits of intensified management scenarios and reduced harvest scenarios depend on the assumed level of substitution. In addition, the magnitude of the deviation from the reference scenario could affect the outcome of such comparisons.

To illustrate these effects, we examine two studies: Petersson et al. (2022) and Gustavsson et al. (2017), both of which compared intensified management with increased area set aside. These studies assumed relatively high substitution levels (1.30 and 0.90–1.1 ton/ton), and in several cases implemented interventions that deviated from recommendations based on practical, economic and ecological considerations.

To evaluate how these assumptions influence the results, we compare the results obtained when using the assumptions made in these studies with the results obtained with a usage-based realized substitution level (RS = 0.53) and with interventions aligned with recommendations from the Swedish Forest Agency.

Figure 4a, which replicates assumptions in Petersson et al. (2022), shows that a 7-fold or 35-fold increase of the fertilized area provide higher climate benefits than doubling the area set aside. The 35-fold increase is not analysed further as it was included by mistake (Petersson et al. 2022). In Fig. 4b the same scenarios are shown but with the substitution level adjusted to the usage-based value (0.53 ton/ton). After a small initial negative effect, increasing the area set aside produces greater climate benefits during the next 120 years.

A similar pattern is seen in Fig. 4c, where the high substitution level is retained, but the intervention levels follow recommended maximum levels. Finally, when combining usage-based substitution with recommended maximum intervention levels, increased set aside area provides considerably greater climate benefits than increased fertilization over the next 150 years (Fig. 4d).

Figure 5 shows a corresponding analysis for scenarios analysed in Gustavsson et al. (2017). Here the reduced-harvest alternative is again a doubling of the area set aside, and the high-production scenario involves a number of actions meant to increase growth and extraction of harvest residues (listed in Table 4). Figure 5a shows that the high-production scenario provides greater climate benefits than doubling the area set aside. Figure 5b shows the same scenarios, but the realized substitution level is lowered to the usage-based value (0.53 ton/ton). Now, increasing the set aside area provides greater climate benefits until 2080, after which the increased production scenario provides greater climate benefits. Retaining the high substitution level, but using recommended maximal intervention levels produces higher climate benefits for increased set a side over the coming 140 years (Fig. 5c). Finally, when both assumptions are adjusted, the set-aside scenario yields much greater climate benefits than the production scenario over at least 150 years (Fig. 5d).

In summary, our analyses suggest that the conclusions that intensified forestry provides greater climate benefits than reduced harvest levels are reversed if the assumed substitution level is based on actual usage of forest biomass and intervention level agree with recommended maximum levels.

## Discussion

Scientific disagreements can impede the development and implementation of important climate policies (Oreskes and Conway 2010). It is crucial, therefore, to resolve the ongoing debate regarding the climate effects of forestry. A first vital step is to identify the different methods and assumptions that lead to conflicting conclusions. This task is often formidable due to the complexities of the analyses, inadequate reporting of methodologies, and variability across different studies. However, the methodological consistency observed within Swedish research on the climate impact of forest management presents a unique opportunity to elucidate “why we disagree”. At the same time, the single-country focus is a limitation, calling for similar investigations across diverse regions.

For the Swedish context our analysis provides a clear answer: By varying the assumed level of substitution and the choice and design of alternative management strategies, one can find support for each of the two sides in the dispute. Consequently, the discussion can now shift towards examining the empirical evidence for various substitution assumptions and exploring which levels of management interventions that are pertinent to the development of climate change mitigation policies.

### Substitution Assumptions

The methodological challenges and uncertainties in calculating displacement factors have been thoroughly discussed by e.g., Howard et al. (2021) and Hurmekoski et al. (2021), who provided a list of best practice methods. Consequently, our focus here will be on aspects relevant for the current debate. Some authors argue that substitution levels may increase in the future due to the development of new products, increased energy efficiency in the industry, increased cascading use and the implementation of carbon capture technologies (Leskinen et al. 2018; Gustavsson et al. 2022; Petersson et al. 2022), whereas others argue for decreasing substitution benefits as fossil fuels and materials are phased out (Harmon 2019; Brunet-Navarro et al. 2021; Myllyviita et al. 2022). Given this uncertainty, we argue that a reasonable strategy is to base estimates of market-level substitution on the present usage of forest biomass. The uncertainty can then be accounted for with sensitivity analyses by specifying hypothetical higher and lower levels (Lundmark et al. 2016; Schulte et al. 2022), or using stochastic simulations (Soimakallio et al. 2016; Niemi et al. 2025). Among the studies we reviewed, those basing their estimates on actual usage reported substitution levels ranging from 0.42 to 0.65. Further support for values in this range is provided by a meta-analysis of market-level displacement factors, which found an average displacement factor of 0.55 (range 0.27–1.16) (Hurmekoski et al. 2021). Even lower values was found in a detailed analysis of substitution in the Finnish forest sector (mean 0.23, range 0.03–0.61) (Niemi et al. 2025). The latter study was based on carbon in harvested wood and considered different societal decarbonisation scenarios that decrease the substitution effect. These values are markedly lower than the mid value utilized by Petersson et al. (2022), who did not provide a clear motivation for the high estimate of 1.3, and Gustavsson et al. (2017) who focused on specific uses (buildings and bio-energy) and used substitution levels in the range 0.9–1.1, based on our estimates.

Some studies (Lundblad et al. 2019; Björheden et al. 2022; Petersson et al. 2022) cite mean values of displacement factors (DF) reported in meta-analyses by Leskinen et al. (2018) (mean DF = 1.2) or Sathre and O’Connor (2010) (mean DF = 2.1). However, these mean values should not be used as market-level displacement factors, as they do not account for the actual usage of biomass for different products. The primary concern is that a majority of the life cycle analyses included in these meta-analyses focuses on products used in the construction sector, which provide high substitution but constitute only a small portion of the biomass used. Thus, while acknowledging the many uncertainties, and that estimates of substitution benefits will evolve with advancing knowledge, we argue that a current best-evidence estimate of realized market level substitution for Swedish conditions is in the range 0.5–0.6.

### Levels of Management Interventions and Choice of Management Strategies

In general, the climate benefits of intensified management strategies increase with the level of intervention, e.g., the extra area fertilized or the extra area used for stump harvest. Thus, it is obvious that the outcome of comparisons of reduced harvest and intensified management will depend on the magnitude of the chosen interventions. This is illustrated by our comparison of the results obtained when using the intervention levels assumed in Petersson et al. (2022) and Gustavsson et al. (2017) and when using intervention levels based on the Forestry Act and recommendations from the Swedish Forest Agency. As the two analyses lead to different conclusions, it is clear that this aspect contributes to the disagreement.

We emphasize that studies of extreme scenarios, such as zero harvest or extensive planting of non-native tree species, can be valuable because they contribute to a more complete understanding of the system’s potential responses. However, it is problematic if the results of such studies are presented to policy makers as relevant for the design of effective policies. Thus, it is important that analyses of extreme scenarios are complemented with analyses of interventions that do not violate the Swedish Forestry Act or its guidelines. Since scientific studies are used to inform policymakers it is crucial that they provide relevant information.

We acknowledge that the concept of “acceptable maximum potential” in forest management can be subjective, and anticipate varying opinions among researchers about what constitutes acceptable harvesting levels, rotation periods, fertilizer use, etc. Therefore, establishing criteria for policy-relevant interventions is essential for achieving consensus. For intensified management practices, we suggest using guidelines provided by the Swedish Forest Agency (Skogsstyrelsen) and the Forestry Research Institute of Sweden (Skogforsk). These guidelines are designed as balanced and feasible compromises between biodiversity preservation, climate impact, economic factors, and practical constraints (Skogsstyrelsen 2018; Skogforsk 2023). Some of those levels, such as the maximum area that can be planted with *Pinus contorta*, are explicitly specified in the Forestry Act and its guidelines. Others are based on more general formulations in the Forestry Act. Currently, there are no equivalent recommendations for acceptable maximum potentials concerning reduced harvests scenarios; the levels we proposed here should be considered an open invitation to further dialogue. It should also be noted that legislations and guidelines are based on political decisions. What is considered acceptable interventions may therefore change over time.

Finally, we note that the management strategy used to reduce harvest may affect the trade-off between short- and long-term effects. The climate benefit is more long-lasting for scenarios with prolonged rotation period and reduced proportion of the growth that is harvested (Fig. 2a, b) than for scenarios where the set-aside area is increased (Fig. 2c). This has implications for the trade-off between biodiversity and climate benefits, as the scenario most beneficial from a biodiversity perspective, i.e., increased set-aside area, is not the best one for mitigating climate warming.

### Limitations of the Analytical Framework

There are important limitations of the analytical framework used in the reviewed studies. One is the poor representation of the tree mortality patterns that we see today. The mortality models used in the studies reviewed here are based on NFI data collected in the period 1983–1992 (Elfving 2014), when mortality rates were considerably lower (by a factor of 4) than those observed during the last 20 years. Storms, drought and pests are believed to be major agents causing increased mortality (Roberge et al. 2024). If this added mortality increases with stand age it will reduce the climate benefit of management actions that leads to increased stand age (Gustavsson et al. 2021; Gustavsson et al. 2022; Petersson et al. 2022). A related issue concerns the need to improve the resilience of the even-aged monocultures that are favoured by today’s forestry. Some studies show that monocultures, especially those of *Picea abies*, are hit particularly hard by storms, drought and pests (Chapin et al. 2007; Valinger and Fridman 2011; de Groot et al. 2019), which suggests that mortality models need to account for differences between even-aged monocultures and mixed forests.

Another important limitation is that market responses are not accounted for. The framework for estimating substitution benefits is based on a static supply perspective (Schulte et al. 2023; Schulte 2024): An increase in the supply of forest products may lead to the substitution of functionally equivalent fossil-based products, but the total product usage, i.e., demand, is assumed to be constant. Thus, the framework is not well suited for analysing scenarios where harvest levels are driven by altered demand, e.g., if harvest levels are modified in response to taxes on biogenic carbon emissions. This is a limitation given that significant reductions in consumption levels are essential to achieve the low-carbon transition envisioned in IPCC’s sustainable development scenarios (IPCC 2021). A related limitation is that carbon leakage is not accounted for. Reduced harvest in one country may lead to increased harvest in other countries (González-Eguino et al. 2017; Lundmark 2022), which may compromise the climate benefit of reduced harvest (Gustavsson et al. 2021; Petersson et al. 2022). This form of leakage is problematic for many national policies, but less so when policies are based on international agreements. A second form of leakage concerns substitution (Liddle 2024). A fundamental assumption when calculating substitution effects is that the substitution of a fossil product with a wood product causes a corresponding amount of fossil carbon to be left in the ground. Logical arguments (Harmon 2019) and econometric analyses of historic national-level data on energy substitution suggest that this assumption is rarely valid (York 2012; Liddle 2024; Rather and Mahalik 2024; Rather et al. 2024). In the future, reduced usage of fossil carbon will likely decrease this form of leakage. However, the same trend will reduce the future benefits of material substitution as the fossil content of products is reduced (Harmon 2019; Brunet-Navarro et al. 2021; Myllyviita et al. 2022). This list of limitations highlights that the estimated climate impact of current forestry practices is likely to evolve as the field progresses. In the Swedish debate reduced harvest strategies are often criticized based on concerns over leakage and higher tree mortality. Because estimates of the effects of these factors are highly uncertain, we speculate that disagreement may persist with a focus on these factors. Thus, there is a need for comprehensive quantitative studies that incorporate these factors.

It is often found that high harvest alternatives provide higher climate benefits than reduced harvest in very long-time perspectives. Thus, it is important that the temporal extent of the simulations is long enough to show how the climate benefits of different strategies change over time. However, the uncertainty increases rapidly with simulation time, both because measurement errors is amplified over time and because factors such as forest growth, management strategies, substitution effects and the use of different biomass fractions is expected to change over time. As the direction of such changes is unknown, we assumed that present conditions will prevail. Moreover, as the reviewed studies used simulation periods ranging from 90 to 200 years, we limited the simulation period to 150 years.

## Conclusions

Our study demonstrates that differing assumptions regarding substitution levels and intervention magnitudes lead to contradictory conclusions about the climate impact of the Swedish forestry. Moving forward, it is essential to establish agreed-upon methods for estimating substitution and formulating management alternatives that are policy-relevant. For analyses of Swedish forestry, we propose i) adopting the Swedish Forest Agency’s guidelines as a basis for setting intervention levels and ii) basing substitution levels on actual biomass use, while also accounting for the uncertainty. Under these assumptions, and given the limitations of the analysis framework outlined above, our analyses suggest that reduced harvest strategies show substantially greater climate benefits in Sweden than both today’s management practices and intensified management over the next 150 years.

## Supplementary information

Supplementary Information

## Data Availability

The Heureka settings, the datasets, Excel calculations and supporting figures are provided as supplementary information in a single file at the journal web page (Supplementary_info_data_calculations_parameters.xlsx).
